# Comparison of predictors of hip fracture and mortality after hip fracture in community-dwellers with and without Alzheimer’s disease – exposure-matched cohort study

**DOI:** 10.1186/s12877-016-0383-2

**Published:** 2016-12-01

**Authors:** Anna-Maija Tolppanen, Heidi Taipale, Antti Tanskanen, Jari Tiihonen, Sirpa Hartikainen

**Affiliations:** 1 School of Pharmacy, University of Eastern Finland, PO Box 1627, 70211 Kuopio, Finland; 2 Research Centre for Comparative Effectiveness and Patient Safety (RECEPS), University of Eastern Finland, PO Box 1627, 70211 Kuopio, Finland; 3 Kuopio Research Centre of Geriatric Care, University of Eastern Finland, PO Box 1627, 70211 Kuopio, Finland; 4 Department of Forensic Psychiatry, Niuvanniemi Hospital, 70240 Kuopio, Finland; 5 Department of Clinical Neuroscience, (CNS), K8, CPF Tiihonen, R5:00, Cpf, Karolinska Universitetssjukhuset Solna 171 76, Stockholm, Sweden; 6 National Institute for Health and Welfare, PO Box 30, 00271 Helsinki, Finland; 7 Department of Psychiatry, Kuopio University Hospital, PO Box 100, 70029 Kuopio, Finland

**Keywords:** Alzheimer’s disease, Hip fracture, Mortality, Cohort study, Risk factors

## Abstract

**Background:**

Dementia, with Alzheimer’s disease (AD) being the most common form, is a major hip fracture risk factor, but currently it is not known whether the same factors predict hip fracture among persons with and without dementia/AD. We compared the predictors of hip fracture and mortality after hip fracture in persons with and without AD.

**Methods:**

An exposure-matched cohort of all community-dwellers of Finland who received a new clinically verified AD diagnosis in 2005–2011 and had no history of previous hip fracture (*N* = 67,072) and an age, sex, and region-matched cohort of persons without AD (*N* = 67,072). Associations between sociodemographic characteristics, comorbidities and medications and risk of hip fracture and mortality after hip fracture were assessed with Cox regression.

**Results:**

As expected, the incidence of hip fractures in 2005–2012 (2.19/100 person-years vs 0.90/100 person-years in the non-AD cohort), as well as mortality after hip fracture (29/100 person-years vs 23/100 person-years in the non-AD cohort) were higher in the AD cohort. This difference was evident regardless of the risk factors. Mental and behavioural disorders (adjusted hazard ratio; HR 95% confidence interval CI: 1.16, 1.09-1.24 and 1.71, 1.52-1.92 in the AD and non-AD-cohorts), antipsychotics (1.12, 1.04-1.20 and 1.56, 1.38-1.76 for AD and non-AD-cohorts) and antidepressants (1.06, 1.00-1.12 and 1.34 1.22-1.47 for AD and non-AD-cohorts) were related to higher, and estrogen/combination hormone therapy (0.87, 0.77-0.9 and 0.79, 0.64-0.98 for AD and non-AD-cohorts) to lower hip fracture risk in both cohorts. Stroke (1.42, 1.26-1.62), diabetes (1.13, 0.99-1.28), active cancer treatment (1.67, 1.22-2.30), proton pump inhibitors (1.14, 1.05-1.25), antiepileptics (1.27, 1.11-1.46) and opioids (1.10, 1.01-1.19) were associated with higher hip fracture risk in the non-AD cohort. Similarly, the associations between mortality risk factors (age, sex, several comorbidities and medications) were stronger in the non-AD cohort.

**Conclusions:**

AD itself appears to be such a significant risk factor for hip fracture, and mortality after hip fracture, that it overrules or diminishes the effect of other risk factors. Thus, it is important to develop and implement preventive interventions that are suitable and effective in this population.

**Electronic supplementary material:**

The online version of this article (doi:10.1186/s12877-016-0383-2) contains supplementary material, which is available to authorized users.

## Background

Alzheimer’s disease (AD), the most common form of dementia, is a key determinant of healthcare costs and health-related quality of life [1]. This is partially due to higher risk of falls and hip fractures among persons with AD [2, 3]. Regardless of cognitive status, hip fracture has been associated with higher mortality, loss of function and mobility [2], but persons with AD have been shown to have an increased risk of death after hip fracture in comparison to those without AD [4]. Similarly, people with dementia have higher risk of institutionalisation or death after hip fracture [5]. Therefore, it is important to identify modifiable risk factors that could be targeted in order to decrease the incidence of hip fractures. As various measures for preventing falls and hip fractures are available [6–11], it is also important to identify non-modifiable risk factors, as they would aid in targeting these interventions to high-risk persons.

Previous studies have shown that women and older individuals have a higher risk of hip fracture [12]. In addition to AD, other comorbidities including previous hip fractures [13], stroke [14], chronic obstructive pulmonary disease (COPD) [15] and other pulmonary diseases [12, 16], diabetes [13, 17], and cardiovascular diseases [12] increase the susceptibility to hip fracture. In addition, various medications such as analgesics, gastroprotectants and different cardiovascular medications, antidepressants, antipsychotics, antiepileptics and COPD medications have been associated with increased hip fracture risk [4, 18, 19]. Similarly, higher age [20] and comorbidities such as cancer, ischaemic heart disease, malignant cancer, diabetes [21, 22] are related to higher mortality after hip fracture. Although women have higher risk of hip fracture, they have been shown to have lower risk of death after hip fracture [20, 21, 23].

However, none of these studies have assessed whether the same factors are associated with hip fracture risk, or mortality after hip fracture among persons with AD, who are particularly susceptible to hip fracture. Therefore, we compared the predictors of hip fracture and mortality after hip fracture in persons with and without AD.

## Methods

### Study cohort

The Medication and Alzheimer’s disease (MEDALZ) cohort includes all community-dwelling persons who received a new clinically verified diagnosis of AD in 2005–2011 (*N* = 70,719) [24]. The age range of the cohort was 34–105 years (mean 80.1 (SD 7.1) years) and 65.2% of the study population were women. Persons with incident AD diagnosis were identified from the Finnish Special Reimbursement Register maintained by the Social Insurance Institution of Finland (SII) as described previously [24]. The Special Reimbursement Register contains records of all persons who are eligible for higher reimbursement due to certain chronic diseases, including AD. To be eligible for reimbursement, the disease must be diagnosed according to specific criterion and diagnosis statement must be submitted to the SII by a physician. The AD diagnosis was mainly based on the National Institute of Neurological and Communicative Disorders and Stroke and the Alzheimer’s Disease and Related Disorders Association’s (NINCDS-ADRDA) and Diagnostic and Statistical Manual of Mental Disorders, 4th Edition (DSM-IV) criteria for Alzheimer’s disease [25, 26]. Briefly, the criterion for AD includes 1) symptoms consistent with mild or moderate AD, 2) decrease in social capacity over a period of at least 3 months, 3) computer tomography (CT)/magnetic resonance imaging scan (MRI) to confirm that neuroanatomical changes are consistent with AD, 4) exclusion of possible alternative diagnoses, and 5) confirmation of the diagnosis by a registered geriatrician or neurologist. Summary of anamnestic information from the patients and family, as well as findings e.g. MRI/CT, laboratory tests, and cognitive tests, are submitted to the SII, where a geriatrician/neurologist systematically evaluates the diagnostic evidence for each AD case and confirms whether the pre-specified criteria are met.

**Fig. 1:**
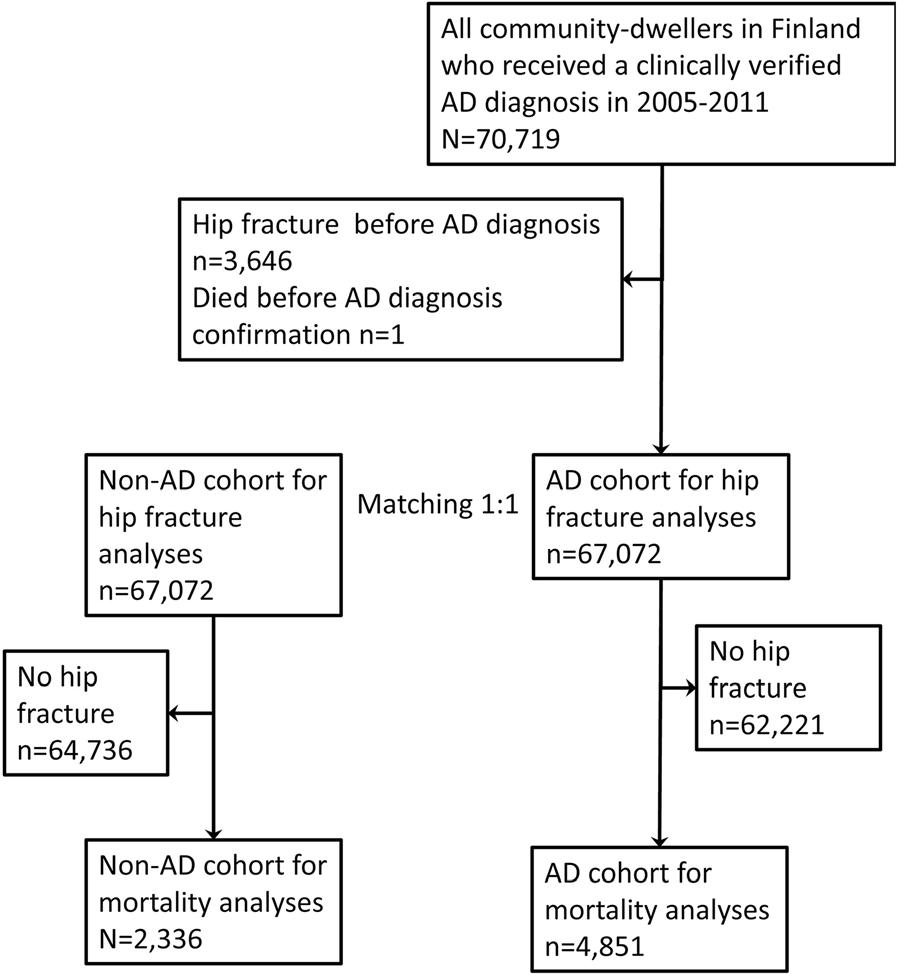
Formation of study samples for hip fracture and mortality risk factor analyses

To compare the hip fracture risk factors and mortality predictors among persons with and without AD, an age, sex- and university hospital district-matched cohort of persons who did not have clinically verified AD diagnosis, was identified from a SII database, which covers all residents of Finland who are eligible for social security. The matching was performed separately for those without previous hip fracture prior to AD diagnosis and those with previous history of hip fracture. Main analyses were restricted to those with no previous hip fracture before the follow-up (*N* = 67,072 in both cohorts, Fig. 1).

For hip fracture analyses, the follow-up for each matched pair began on the date of AD diagnosis of the index case and ended on the date of first incident hip fracture during the study period, date of death or end of the follow-up (December 31, 2012), whichever occurred first. For mortality predictors, the follow-up began on the date of first incident hip fracture during the follow-up and ended on date of death or end of follow-up.

Each resident of Finland is assigned a unique personal identity code which was used to compile the research database from various national registers as described previously [27]. All data were de-identified (i.e., the personal identity codes were substituted by anonymous numerical codes) by the register maintainers before the data were submitted to the research team. Study participants were not contacted. According to the Finnish legislation, ethics committee approval or informed consent were not required. The study protocol was approved by the register maintainers (Statistics Finland, SII and National Institute of Health and Welfare).

### Hip fracture and mortality after hip fracture

Hip fractures during 1972–2012 were identified from the national Care Register for Health Care, based on the International Classification of Diseases (ICD) with following ICD-10 codes (and corresponding ICD-9 and ICD-8 codes): S72.0 (fracture of neck of femur), S72.1 (pertrochanteric fracture) and S72.2 (subtrochanteric fracture). Finnish Care Register for Health Care is a statutory register containing information on use of in- and outpatient healthcare services. The individual-level data are collected and updated continuously. Our study was restricted to inpatient part of the register. A study comparing audit and register-based data showed that 98.1% of occurred hip fractures were recorded in the inpatient data of the Care Register for Health Care [28]. Mortality data (2005–2012) were obtained from the Causes of Death Register, maintained by Statistics Finland.

### Predictors

Socioeconomic position (SEP), defined by the occupational social class, was obtained from the censuses maintained by Statistics Finland. The data were collected on five-year intervals between 1970 and 1990, on 1993, 1995, 2000, and annually from 2004 onwards. The 2010 version of the original classification is available from reference http://www.stat.fi/meta/luokitukset/ammatti/001-2010/index_en.html, older versions available from authors by request). An ordinal variable with the following categories was derived “managerial/professional”, “office worker”, “farming/forestry”, “sales/industry/cleaning”, “unknown” and “did not respond”. For each individual, the highest class from 1970 until the beginning of the follow-up was used.

History of comorbidities before the follow-up (since 1972) were identified from the National Hospital Discharge Register and Special Reimbursement Register. Data on diabetes, cardiovascular diseases (hypertension, coronary artery disease, familial hypercholesterolemia, heart failure, and chronic cardiac arrhythmias) and asthma or chronic obstructive pulmonary disease were obtained from the Special Reimbursement Register. Data on stroke (ICD-10 codes I60-I64), and mental and behavioural disorders (ICD-10 codes F*) were obtained from the National Hospital Discharge Register. In addition, active cancer treatment within five years before the follow-up was defined from the Hospital Discharge register (Nordic Medico-Statistical Committee procedure codes for radiotherapy; AAG50, AX, HA0, PJO, QA0, QB0, QC0, QD0, QW0, QX0, WA, WB, WC, WD, WE, WF0, WFO and ZX0) and Prescription Register (antineoplastic drugs with the following ATC-codes: L01, L02, L03AA, L03AB01, L03AB04, L03AB05, L03AC, L03AX, L04AA10, L04AA34, L04AA18, L04AX02, L04AX03 excluding L01BA01 and L04AX03 users with rheumathoid arthritis). Prosthetic hip and knee replacements were identified with the Finnish version of the Nordic Medico-Statistical Committee (NOMESCO) classification for Surgical Procedures (NCSP) using the following codes: NFB and NFC for hip joint and NGB, NGC for knee.

Medication use was defined as medication use within the previous five years before the date of cohort entry. For mortality analyses, the time window was five years before hip fracture date. Data on medication use were obtained from the Finnish National Prescription Register which covers reimbursed prescription drug purchases. All drugs in the Prescription Register are categorised according to the World Health Organization (WHO) Anatomical Therapeutic Chemical (ATC) Classification system and purchased amounts are recorded in Defined Daily Doses (DDDs); the assumed average maintenance dose per day for a drug used for its main indication in adults [29]. For each person, the drug use periods for each ATC code have been modelled with a validated PRE2DUP (From prescription drug purchases to drug use periods) method [30]. PRE2DUP is based on sliding averages of DDD and it accounts for hospitalisations, stockpiling and dose changes.

Medications were identified with the following ATC-codes; use of any cardiovascular drug (C*), drugs for obstructive airway diseases (ATC code R03), anti-Parkinson drugs (N04), estrogen and estrogen-progesterone combinations (G03C and G03F), bisphosphonates (M05BA, M05BB), proton pump inhibitors (A02BC), antiepileptic drugs (N03), antipsychotics (N05A excluding lithium (N05AN)), antidepressants (N06A), benzodiazepines and benzodiazepine-related drugs (BZDRs; N05BA, N05CD and N05CF) and opioids (N02A).

### Statistical analyses

All statistical analyses were performed with Stata/MP 14.1. Characteristics between AD and non-AD cohorts were compared with *t*-test and *χ* ^2^-test. Associations between predictors and hip fracture, and predictors and death after hip fracture were assessed with Cox regression. Proportional hazards assumptions were confirmed with Schoenfeld residuals. Main analyses were restricted to those without previous hip fracture before cohort entry, but additional sensitivity analyses among those with previous hip fracture are presented in Additional file 1: Table S1–S3. These analyses were conducted to assess whether the associations were similar when persons with previous hip fractures were included.

## Results

### Hip fracture risk factors

**Fig. 2:**
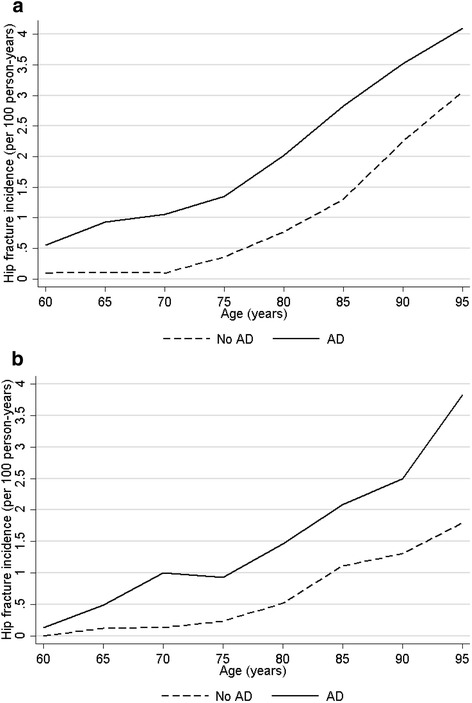
Incidence of hip fracture in **a** women and **b** men in persons with and without Alzheimer’s disease. Due to data sparsity in the youngest and oldest age groups, the age range was restricted to 60–95 years

The follow-up time was 231,939 person-years in the AD cohort and 269,905 person-years in the non-AD cohort. The average follow-up in the AD cohort was 3.0 years (range 1 day-8.0 years) and in the non-AD cohort 3.6 years (range 1 day-8.0 years). Altogether 5,264 hip fractures occurred in the AD cohort (incidence rate 2.23 hip fractures/100 person-years). In the non-AD cohort, the incidence rate was 0.98 hip fractures/100 person-years (n of fractures = 2,643). When persons with previous fractures were excluded the incidence rates were 2.19 in the AD cohort (n of hip fractures = 4,851) and 0.90 in the non-AD cohort (n of hip fractures = 2,336). Persons with AD had higher relative risk of hip fracture during the follow-up (SEP-adjusted HR 2.35, 95% CI 2.24-2.46 when those with previous fractures were included, HR = 2.46, 95% CI 2.34-2.59 when they were excluded). The impact of AD on hip fracture risk is illustrated in Fig. 2, which shows that although the risk of hip fracture increases steadily across aging in both AD and non-AD cohorts (in both sexes) and the changes occur in parallel, the incidence rate in the AD cohort remains constantly higher.

Characteristics of AD and non-AD cohort are shown in Table 1 and characteristics of those with previous hip fracture in Additional file 1: Table S1. Persons in the AD cohort experienced their hip fracture at younger age than in the non-AD cohort. Cardiovascular diseases (CVD), stroke, diabetes, hip and knee replacements and any mental or behavioural disorder were more common in the AD cohort. They were also more likely to have CVD medication, use bisphosphonates, anti-Parkinson drugs, antiepileptics, antipsychotics, antidepressants, proton pump inhibitors, BZDRs or opioids than those without AD. The differences were similar among those with a previous hip fracture.

**Table 1:** Characteristics of AD and non-AD cohorts

Characteristic	AD cohort *N* = 67,072	Non-AD cohort *N* = 67,072	*P*
Age at baseline, mean (95% CI)	79.9 (79.8–79.9)	79.9 (79.8–79.9)	matched
Age at first fracture during the follow-up	84.1 (83.9–84.3)	86.3 (86.1–86.5)	<0.001
Sex			
Men	43158 (64.3)	43158 (64.3)	matched
Women	23914 (35.7)	23914 (35.7)	
Highest occupational social class before follow-up			
Managerial/professional	14072 (21.0)	14454 (21.5)	<0.001
Office worker	5608 (8.4)	5629 (8.4)	
Farming/forestry	12735 (19.0)	13160 (19.6)	
Sales/industry/cleaning	28669 (42.7)	26180 (39.0)	
Unknown	5470 (8.2)	5186 (7.7)	
Did not respond	518 (0.8)	2463 (3.7)	
Hip replacement	2219 (3.3)	1001 (1.5)	<0.001
Knee replacement	105 (0.2)	67 (0.1)	0.004
Cardiovascular disease	34046 (50.8)	32841 (49.0)	<0.001
Stroke	6356 (9.5)	5232 (7.8)	<0.001
Diabetes	8969 (13.4)	7527 (11.2)	<0.001
Asthma/COPD	5846 (8.7)	6011 (9.0)	0.11
Cancer treatment	792 (1.2)	855 (1.3)	0.12
Any mental or behavioural disorder	15365 (22.9)	5917 (8.8)	<0.001
Any CVD medication	57603 (85.9)	54704 (81.6)	<0.001
Drugs for obstructive airway diseases	15895 (23.7)	15991 (23.8)	0.54
Hormone therapy use	4429 (6.6)	4213 (6.3)	0.16
Bisphosphonates	8932 (13.3)	7460 (11.1)	<0.001
Proton pump inhibitors	22281 (33.2)	21760 (32.4)	0.002
Anti-Parkinson drugs	2349 (3.5)	1959 (2.9)	<0.001
Antiepileptics drugs	6729 (10.0)	5697 (8.5)	<0.001
Antipsychotics	11127 (16.6)	5364 (8.0)	<0.001
Antidepressants	23044 (34.4)	13821 (20.6)	<0.001
Benzodiazepines and related drugs	29368 (43.8)	25915 (38.6)	<0.001
Opioids	28907 (43.1)	25742 (38.4)	<0.001

Data are given as n (%) unless otherwise indicated

Regardless of risk factor category, the age- and sex-adjusted incidence rate of hip fracture were higher in the AD cohort (Tables 2 and 3). Higher age was associated with higher risk of hip fracture in both cohorts, but the association was stronger in the non-AD cohort. In general, the associations of sex and occupational social class were of similar magnitude in both cohorts, with the exception of the ‘Unknown’ category, that had lower risk of hip fracture in the non-AD cohort. After adjusting for occupational social class, history of mental and behavioural disorders, having a hip or knee replacement, use of antipsychotics and antidepressants or anti-Parkinson drugs were related to higher risk, and hormone therapy to lower hip fracture risk in both cohorts. Stroke, diabetes, active cancer treatment, proton pump inhibitors, antiepileptics, BZDRs and opioids were associated with higher hip fracture risk in the non-AD cohort only, while any CVD medication was associated with lower risk in the AD cohort. The results remained similar when those with previous hip fracture were included (Additional file 1: Table S2). Previous hip fracture was related to higher risk of hip fracture during the follow-up and as with other comorbidities and medications, the association was stronger in those without AD.

**Table 2:** Hip fracture risk factors (demographic and comorbidities) in AD and non-AD cohorts (persons with previous fractures excluded)

Risk factor	Category	AD cohort			Non-AD cohort		
		IR/ 100 PY	Unadjusted HR (95% CI)	Adjusted HR^a^ (95% CI)	IR/ 100 PY	Unadjusted HR HR (95% CI)	Adjusted HR^a^ HR (95% CI)
Age at baseline, increase per year			1.07 (1.06–1.07)	1.06 (1.06–1.07)	N.A	1.12 (1.11–1.12)	1.12 (1.11–1.13)
Sex	Men	2.50	1.00 (reference)	1.00 (reference)	1.05	1.00 (reference)	1.00 (reference)
	Women	1.58	0.63 (0.59–0.68)	0.73 (0.68–0.78)	0.64	0.61 (0.56–0.67)	0.76 (0.69–0.84)
Highest occupational social class before follow-up	Managerial/professional	1.83	1.00 (reference)	1.00 (reference)	0.66	1.00 (reference)	N.A
	Office worker	2.52	1.38 (1.23–1.54)	1.17 (1.04–1.31)	0.95	1.45 (1.23–1.72)	
	Farming/forestry	2.05	1.12 (1.02–1.24)	0.95 (0.86–1.04)	0.94	1.45 (1.26–1.66)	
	Sales/industry/cleaning	2.15	1.18 (1.08–1.27)	1.04 (0.96–1.13)	0.96	1.47 (1.31–1.66)	
	Unknown	3.27	1.79 (1.62–1.99)	1.19 (1.06–1.32)	1.48	2.30 (1.97–2.68)	
	Did not respond	2.50	1.36 (1.00–1.87)	1.16 (0.85–1.58)	0.25	0.38 (0.25–0.56)	
Hip replacement	No	1.22	1.00 (reference)	1.00 (reference)	0.52	1.00 (reference)	1.00 (reference)
	Yes	41.83	42.0 (39.5–44.6)	38.8 (36.5–41.2)	36.47	94.3 (86.4–103.0)	71.2 (65.1–77.9)
Knee replacement	No	2.14	1.00 (reference)	1.00 (reference)	0.88	1.00 (reference)	1.00 (reference)
	Yes	59.15	28.4 (23.4–34.4)	25.3 (20.8–30.7)	36.84	45.9 (35.9–58.6)	40.3 (31.5–51.5)
Cardiovascular disease	No	2.18	1.00 (reference)	1.00 (reference)	0.83	1.00 (reference)	1.00 (reference)
	Yes	2.19	1.01 (0.95–1.06)	0.94 (0.89–1.00)	0.98	1.20 (1.11–1.30)	0.99 (0.91–1.07)
Stroke	No	2.18	1.00 (reference)	1.00 (reference)	0.86	1.00 (reference)	1.00 (reference)
	Yes	2.30	1.06 (0.96–1.17)	1.05 (0.95–1.16)	1.42	1.67 (1.47–1.89)	1.42 (1.26–1.62)
Diabetes	No	2.19	1.00 (reference)	1.00 (reference)	0.89	1.00 (reference)	1.00 (reference)
	Yes	2.14	0.98 (0.90–1.06)	1.03 (0.95–1.13)	0.99	1.11 (0.98–1.26)	1.13 (0.99–1.28)
Cancer treatment	No	2.19	1.00 (reference)	1.00 (reference)	0.90	1.00 (reference)	1.00 (reference)
	Yes	2.06	0.94 (0.71–1.24)	1.01 (0.76–1.34)	1.31	1.44 (1.05–1.98)	1.67 (1.22–2.30)
Asthma/COPD	No	2.18	1.00 (reference)	1.00 (reference)	0.90	1.00 (reference)	1.00 (reference)
	Yes	2.24	1.02 (0.93–1.13)	1.03 (0.94–1.14)	0.95	1.05 (0.91–1.21)	1.07 (0.93–1.23)
Any mental or behavioural disorder	No	2.09	1.00 (reference)	1.00 (reference)	0.85	1.00 (reference)	1.00 (reference)
	Yes	2.53	1.21 (1.14–1.29)	1.16 (1.09–1.24)	1.53	1.83 (1.63–2.05)	1.71 (1.52–1.92)

^a^Adjusted for occupational social class

**Table 3:** Hip fracture risk factors (medications) in AD and non-AD cohorts (persons with previous fractures excluded)

Risk factor	Category	AD cohort			Non-AD cohort		
		IR/ 100 PY	Unadjusted HR (95% CI)	Adjusted HR^a^ (95% CI)	IR/ 100 PY	Unadjusted HR HR (95% CI)	Adjusted HR^a^ HR (95% CI)
Any CVD medication	No	2.21	1.00 (reference)	1.00 (reference)	0.63	1.00 (reference)	1.00 (reference)
	Yes	2.18	0.99 (0.91–1.06)	0.84 (0.78–0.91)	0.98	1.54 (1.37–1.73)	1.02 (0.91–1.15)
Drugs for obstructive airway diseases	No	2.21	1.00 (reference)	1.00 (reference)	0.90	1.00 (reference)	1.00 (reference)
	Yes	2.11	0.96 (0.89–1.02)	0.97 (0.90–1.03)	0.92	1.01 (0.92–1.11)	1.00 (0.91–1.11)
Hormone therapy	No	2.23	1.00 (reference)	1.00 (reference)	0.93	1.00 (reference)	1.00 (reference)
	Yes	1.66	0.74 (0.66–0.84)	0.87 (0.77–0.98)	0.51	0.54 (0.44–0.67)	0.79 (0.64–0.98)
Bisphosphonates	No	2.13	1.00 (reference)	1.00 (reference)	0.88	1.00 (reference)	1.00 (reference)
	Yes	2.58	1.21 (1.12–1.31)	0.97 (0.90–1.05)	1.14	1.29 (1.14–1.46)	0.96 (0.85–1.08)
Proton pump inhibitors	No	2.18	1.00 (reference)	1.00 (reference)	0.83	1.00 (reference)	1.00 (reference)
	Yes	2.19	1.00 (0.94–1.07)	0.96 (0.90–1.02)	1.07	1.27 (1.17–1.39)	1.14 (1.05–1.25)
Anti-Parkinson drugs	No	2.16	1.00 (reference)	1.00 (reference)	0.88	1.00 (reference)	1.00 (reference)
	Yes	2.95	1.36 (1.19–1.57)	1.46 (1.27–1.68)	1.66	1.86 (1.53–2.26)	1.67 (1.38–2.03)
Antiepileptics drugs	No	2.20	1.00 (reference)	1.00 (reference)	0.88	1.00 (reference)	1.00 (reference)
	Yes	2.09	0.95 (0.86–1.05)	0.99 (0.90–1.09)	1.23	1.37 (1.19–1.57)	1.27 (1.11–1.46)
Antipsychotics	No	2.12	1.00 (reference)	1.00 (reference)	0.85	1.00 (reference)	1.00 (reference)
	Yes	2.53	1.20 (1.11–1.29)	1.12 (1.04–1.20)	1.64	1.95 (1.73–2.20)	1.56 (1.38–1.76)
Antidepressants	No	2.14	1.00 (reference)	1.00 (reference)	0.83	1.00 (reference)	1.00 (reference)
	Yes	2.28	1.07 (1.00–1.13)	1.06 (1.00–1.12)	1.24	1.49 (1.36–1.64)	1.34 (1.22–1.47)
BZDRS	No	2.04	1.00 (reference)	1.00 (reference)	0.74	1.00 (reference)	1.00 (reference)
	Yes	2.39	1.17 (1.11–1.24)	1.03 (0.97–1.09)	1.18	1.58 (1.45–1.71)	1.22 (1.12–1.32)
Opioids	No	2.09	1.00 (reference)	1.00 (reference)	0.80	1.00 (reference)	1.00 (reference)
	Yes	2.34	1.12 (1.05–1.18)	1.00 (0.94–1.06)	1.12	1.36 (1.25–1.48)	1.10 (1.01–1.19)

^a^Adjusted for occupational social class

### Predictors of mortality after hip fracture

The median follow-up time after hip fracture was 1.3 years (range 1 day-7.7 years) in the AD cohort and 1.2 years (range 1 day-7.2 years) in the non-AD cohort. The average follow-up from hip fracture to death was 261 days (range 1 day-7.6 years) in the AD cohort and 212 days (range 1 day-7.6 years) in the non-AD cohort. Altogether 2,407 persons with AD (49.6%) died after experiencing a hip fracture, in comparison to non-AD cohort in which 923 persons (39.5%) of those who experienced a hip fracture died. Persons with AD had higher relative risk of death after hip fracture (SEP-adjusted HR 1.41, 95% CI 1.31-1.52). The excess risk was similar in men and women (P for interaction 0.76), but more pronounced among those who were diagnosed AD at younger age (or experienced the first hip fracture at younger age) (P for interaction <0.001). The association was stronger among those who were <85 years old (SEP-adjusted HR 1.72, 95% CI 1.52-1.95) than among those who were at least 85 years old when they experienced the fracture (adjusted HR 1.33, 95%CI 1.22-1.46).

Women and older individuals had higher risk of death after hip fracture in both cohorts, while occupational social class was not associated with mortality after hip fracture (Table 4). History of CVD, stroke, mental and behavioural disorders, CVD medication, proton pump inhibitors, antipsychotics and opioids were associated with higher mortality risk in both cohorts but the associations were stronger in the non-AD cohort (Tables 4 and 5). Antidepressants, BZDRs, and cancer treatment were associated with higher mortality risk in the non-AD group while diabetes, asthma/COPD, drugs for obstructive airway diseases and antiepileptic drugs were related to higher mortality in the AD cohort. Persons with knee replacement had lower risk of death in the AD cohort. Similar associations were observed when those with previous hip fracture were included in the analyses (Additional file 1: Table S3), although the association between cancer treatment and mortality in the non-AD cohort was no longer evident.

**Table 4:** Predictors of mortality (demographics and comorbidities) after hip fracture (persons with previous fractures excluded)

Risk factor	Category	AD cohort			Non-AD cohort		
		Mortality/ 100 PY	Unadjusted HR (95% CI)	Adjusted HR^a^ (95% CI)	Mortality/ 100 PY	Unadjusted HR (95% CI)	Adjusted HR^a^ (95% CI)
Age at baseline, increase per year			1.04 (1.03–1.05)	1.05 (1.05–1.06)	N.A	1.07 (1.06–1.09)	1.08 (1.07–1.10)
Sex	Men	25.1	1.00 (reference)	1.00 (reference)	19.4	1.00 (reference)	1.00 (reference)
	Women	47.7	1.90 (1.74–2.08)	2.09 (1.90–2.29)	39.3	2.06 (1.79–2.36)	2.43 (2.10–2.80)
Highest occupational social class before follow-up	Managerial/professional	29.9	1.00 (reference)	1.00 (reference)	23.4	1.00 (reference)	N.A
	Office worker	25.6	0.85 (0.72–1.01)	1.02 (0.86–1.21)	20.7	0.88 (0.66–1.16)	
	Farming/forestry	30.6	1.02 (0.89–1.17)	1.01 (0.88–1.15)	23.0	0.97 (0.78–1.21)	
	Sales/industry/cleaning	29.6	0.99 (0.88–1.11)	1.05 (0.93–1.18)	23.7	1.00 (0.83–1.22)	
	Unknown	28.8	0.96 (0.83–1.12)	1.04 (0.89–1.21)	24.5	1.05 (0.82–1.34)	
	Did not respond	25.7	0.86 (0.55–1.33)	1.03 (0.67–1.61)	23.3	0.97 (0.48–1.98)	
Hip replacement	No	29.36	1.00 (reference)	1.00 (reference)	23.38	1.00 (reference)	1.00 (reference)
	Yes	21.85	0.74 (0.49–1.13)	0.68 (0.45–1.04)	20.36	0.86 (0.46–1.61)	1.22 (0.65–2.29)
Knee replacement	No	29.62	1.00 (reference)	1.00 (reference)	23.47	1.00 (reference)	1.00 (reference)
	Yes	19.88	0.67 (0.52–0.87)	0.73 (0.56–0.95)	18.83	0.80 (0.51–1.24)	0.88 (0.56–1.38)
Cardiovascular disease	No	26.5	1.00 (reference)	1.00 (reference)	20.2	1.00 (reference)	1.00 (reference)
	Yes	32.1	1.21 (1.12–1.32)	1.19 (1.10–1.29)	26.0	1.28 (1.12–1.47)	1.31 (1.15–1.50)
Stroke	No	28.7	1.00 (reference)	1.00 (reference)	22.3	1.00 (reference)	1.00 (reference)
	Yes	36.8	1.28 (1.12–1.47)	1.19 (1.03–1.37)	37.9	1.70 (1.38–2.09)	1.67 (1.35–2.06)
Diabetes	No	28.7	1.00 (reference)	1.00 (reference)	23.3	1.00 (reference)	1.00 (reference)
	Yes	33.3	1.16 (1.03–1.31)	1.22 (1.08–1.38)	23.9	1.03 (0.85–1.24)	1.17 (0.96–1.42)
Asthma/COPD	No	28.9	1.00 (reference)	1.00 (reference)	23.5	1.00 (reference)	1.00 (reference)
	Yes	33.4	1.16 (1.01–1.33)	1.17 (1.02–1.34)	22.2	0.93 (0.74–1.17)	1.04 (0.83–1.31)
Cancer treatment	No	29.3	1.00 (reference)	1.00 (reference)	23.3	1.00 (reference)	1.00 (reference)
	Yes	23.6	0.81 (0.52–1.24)	0.88 (0.57–1.35)	26.4	1.12 (0.68–1.83)	1.63 (0.99–2.68)
Any mental or behavioural disorder	No	28.2	1.00 (reference)	1.00 (reference)	22.8	1.00 (reference)	1.00 (reference)
	Yes	31.6	1.12 (1.03–1.22)	1.18 (1.08–1.29)	28.1	1.25 (1.02–1.54)	1.28 (1.04–1.58)

^a^Adjusted for occupational social class

**Table 5:** Predictors of mortality (medications) after hip fracture in AD and non-AD cohorts (persons with previous fractures excluded)

Risk factor	Category	AD cohort			Non-AD cohort		
		Mortality/ 100 PY	Unadjusted HR (95% CI)	Adjusted HR^a^ (95% CI)	Mortality/ 100 PY	Unadjusted HR (95% CI)	Adjusted HR^a^ (95% CI)
Any CVD medication	Yes	31.6	1.12 (1.03–1.22)	1.18 (1.08–1.29)	28.1	1.25 (1.02–1.54)	1.28 (1.04–1.58)
	No	24.7	1.00 (reference)	1.00 (reference)	17.4	1.00 (reference)	1.00 (reference)
	Yes	30.0	1.17 (1.05–1.31)	1.17 (1.04–1.31)	24.0	1.36 (1.06–1.74)	1.37 (1.07–1.76)
Drugs for obstructive airway diseases	No	28.3	1.00 (reference)	1.00 (reference)	23.1	1.00 (reference)	1.00 (reference)
	Yes	35.3	1.25 (1.12–1.39)	1.17 (1.05–1.30)	24.3	1.04 (0.88–1.23)	1.11 (0.94–1.32)
Hormone therapy	No	29.8	1.00 (reference)	1.00 (reference)	23.7	1.00 (reference)	1.00 (reference)
	Yes	18.6	0.63 (0.49–0.80)	0.81 (0.64–1.04)	11.7	0.50 (0.29–0.85)	0.78 (0.46–1.32)
Bisphosphonates	No	29.5	1.00 (reference)	1.00 (reference)	23.7	1.00 (reference)	1.00 (reference)
	Yes	28.1	0.95 (0.85–1.07)	1.07 (0.95–1.20)	21.0	0.88 (0.72–1.06)	1.06 (0.87–1.29)
Proton pump inhibitors	No	26.8	1.00 (reference)	1.00 (reference)	21.0	1.00 (reference)	1.00 (reference)
	Yes	33.4	1.24 (1.15–1.35)	1.25 (1.15–1.36)	26.8	1.27 (1.12–1.45)	1.31 (1.15–1.49)
Anti–Parkinson drugs	No	29.28	1.00 (reference)	1.00 (reference)	23.12	1.00 (reference)	1.00 (reference)
	Yes	29.10	0.99 (0.84–1.18)	1.02 (0.86–1.21)	27.15	1.18 (0.91–1.54)	1.20 (0.92–1.56)
Antiepileptics drugs	No	29.0	1.00 (reference)	1.00 (reference)	23.6	1.00 (reference)	1.00 (reference)
	Yes	31.9	1.10 (0.96–1.25)	1.12 (0.99–1.28)	21.2	0.90 (0.72–1.11)	0.98 (0.79–1.21)
Antipsychotics	No	28.2	1.00 (reference)	1.00 (reference)	22.3	1.00 (reference)	1.00 (reference)
	Yes	31.1	1.10 (1.01–1.20)	1.16 (1.06–1.26)	30.9	1.41 (1.18–1.68)	1.41 (1.18–1.68)
Antidepressants	No	30.7	1.00 (reference)	1.00 (reference)	22.1	1.00 (reference)	1.00 (reference)
	Yes	27.5	0.89 (0.82–0.97)	0.97 (0.89–1.05)	26.9	1.22 (1.05–1.40)	1.29 (1.11–1.48)
BZDRS	No	28.4	1.00 (reference)	1.00 (reference)	21.3	1.00 (reference)	1.00 (reference)
	Yes	30.2	1.06 (0.98–1.15)	1.06 (0.98–1.15)	25.7	1.21 (1.06–1.38)	1.18 (1.04–1.35)
Opioids	No	25.5	1.00 (reference)	1.00 (reference)	18.7	1.00 (reference)	1.00 (reference)
	Yes	32.9	1.30 (1.20–1.41)	1.29 (1.19–1.40)	28.5	1.50 (1.31–1.72)	1.56 (1.36–1.79)

^a^Adjusted for occupational social class

## Discussion

Our findings indicate that in general, the same risk factors were associated with hip fracture risk in persons with and without AD, although the associations were stronger among those without AD. Similar results were observed with mortality predictors, with the exception of knee replacements that were not associated with mortality in the non-AD cohort. This different association likely reflects patient selection for knee replacement among persons with AD. One possible explanation is that AD itself is such a strong risk factor for hip fracture [3] and adverse outcomes after hip fracture [4], that it overrules the impact of other comorbidities and risk factors. This was illustrated in the hip fracture incidence rate graphs: for example, the hip fracture risk of a 75-year-old woman with AD was equal to that of an 85-year-old women without AD. To our knowledge, this is the first study assessing the predictors of hip fracture and mortality after hip fracture among persons with AD/dementia and comparing the magnitude of these associations to those without AD. As the aim was to compare the relative risk associated with individual predictors, the results were not adjusted for comorbidity index. This would have also lead into severe collinearity issues as many of the predictors (such as stroke, diabetes and cancer) are included in most indices.

Strengths of our study include a nationwide cohort of persons with clinically verified AD diagnosis and accurately recorded hip fractures [31] and mortality. Thus, there was no loss to follow-up bias. Studies assessing the internal validity of Finnish Care Register for Health Care and comparing register information with patient records or other information from the primary source have confirmed that the coverage and accuracy of these registers are well-suited for epidemiological research (reviewed in reference [32]. The agreement between self-reported drug use interview and drug use periods modelled from the prescription register was very good or good for cardiovascular drugs, drugs for obstructive airway diseases, bisphosphonates, proton pump inhibitors, psychotropics and opioids [33]. Hormone therapy, antiepileptics and anti-Parkinson drugs were not included in that study, but as these are mainly used regularly, and good or very good agreement was observed for regularly used drugs [33], exposure to them should also be captured from the prescription register. We were also able to compare the associations to persons without AD. Our sample was not selected on the basis of socioeconomic position, which increases the generalisability of our findings.

Our study is limited in the sense that the sample was restricted to those who were community-dwelling at the beginning of follow-up. Therefore the results are not entirely generalisable to institutionalised persons. This restriction was applied because medications provided at certain institutions are not recorded in the prescription register and thus inclusion of institutionalised persons would have increased the possibility of misclassification bias. The data were obtained from national registers, which include all purchased reimbursable medications. Although purchased medications may not always reflect consumed medications, our results are not prone to recall bias, and the dispensing data approximates the medication use better than prescription data. Further, data on risk factors such as smoking, bone mass density or weight changes were not available and thus it would be important to assess the impact of these risk factors in future studies.

Similar to previous studies and meta-analyses, women and older individuals had higher risk of hip fracture [12] while men had higher mortality after experiencing a hip fracture in both cohorts [20, 21, 23]. Stroke and cancer (indicated by radiotherapy or antineoplastic medication) were associated with higher risk of hip fracture in the non-AD cohort only. In addition, there was a tendency for increased hip fracture risk among those with diabetes in the non-AD cohort, but the confidence intervals included also 1. Mental and behavioural disorders (i.e., the entire F chapter of ICD-10) were associated with higher risk in both cohorts. This could in part be due to the association of psychotropic medications, as observed in ours and previous studies [4, 18]. Alternatively, it may also reflect the association between dementia and hip fracture. Consistent with previous studies [4, 18, 19], antidepressant and antipsychotic use were related to higher risk of hip fracture in both cohorts and BZDRs with hip fracture risk [34, 35] in the non-AD cohort.

The majority of the identified risk factors, such as sex, age, cancer treatment, CVD, stroke and psychiatric illness can essentially be considered as non-modifiable. However, our result highlight the overall impact of preventive efforts on e.g. dementia and cardiovascular diseases, as the burden of these diseases is not restricted to the condition *per se*, because they may further predispose persons to other comorbidities such as hip fracture. Similarly, some of the identified medications may be non-modifiable, as these medications may be inevitably needed by the patient. However, psychotropic medications impair cognition and balance [36] and increase sedative load [37], thus doubling the risk of falls among older persons [38, 39].

Different preventive interventions, for falls [6] and fall-related injuries [7, 8] have been proposed, including exercise, medications, home safety assessment and modification interventions, as well as medication reviews. However, majority of these have focussed on community-dwellers in general [7, 8, 11] and thus they may not be directly applicable to persons with dementia, or their uptake or effectiveness may be different in this population. As the impact of AD in hip fracture risk appears to exceed the impact of other hip fracture risk factors, it would be important to develop and implement measures that can be applied to this population group. Regular medication reviews are one feasible approach. Psychotropic medications increase the hip fracture risk, and they are also widely used by persons with AD [40–42]. Thus, reducing the prescription of psychotropic medications by either seeking non-pharmacological alternatives to their use in the first place, or, for patients for whom these medications are deemed necessary, regular monitoring and discontinuation efforts are examples of possible preventive measures that are hypothesised to decrease the incidence of falls in older population [39]. Recent systematic review suggested that antipsychotic medication can be withdrawn from many persons with AD and behavioural and psychological symptoms of dementia without detrimental effects on their behaviour, although withdrawal might not be recommended for those with most severe symptoms [43].

## Conclusions

Our results may indicate that AD itself is such a significant hip fracture risk factor that it overrules or diminishes the effect of other risk factors. Therefore, it would be important to develop and implement preventive measures that are suitable for persons with AD. Further, as our study was restricted to persons who were community-dwelling at baseline, future studies among institutionalised persons are needed.

## Additional file

Additional file 1: Supplementary Tables S1-S3. (DOCX 29.4 kb)

## Abbreviations

AD: Alzheimer’s disease
ATC: Anatomical therapeutic chemical-classification system
BZDR: Benzodiazepines and benzodiazepine-related drugs
CT: Computer tomography
CVD: Cardiovascular diseases
DSM-IV: Diagnostic and statistical manual of mental disorders, 4th Edition
ICD: International classification of diseases
MEDALZ: The medication and alzheimer’s disease study
MRI: Magnetic resonance imaging scan
NINCDS-ADRDA: Alzheimer’s disease and related disorders sssociation
PRE2DUP: From prescription drug purchases to drug use periods-method
SEP: Socioeconomic position
SII: Social insurance institution of Finland
WHO: World Health Organization
