# Supplementary material for: Comparison of predictors of hip fracture and mortality after hip fracture in community-dwellers with and without Alzheimer’s disease – exposure-matched cohort study
Source: BMC Geriatr. 2016 Dec 1;16:204. doi: 10.1186/s12877-016-0383-2 (PMC5134120; doi:10.1186/s12877-016-0383-2)
Supplement: Additional file 1: — Supplementary Tables S1-S3. (DOCX 29.4 kb) [file 12877_2016_383_MOESM1_ESM.docx]

**Table S1 Characteristics persons with previous fractures in the AD and non-AD cohorts. Data are given as n (%) unless otherwise indicated. Abbreviations: COPD; chronic obstructive pulmonary disease; CVD cardiovascular disease; PPI proton pump inhibitors; BZDRs benzodiazepines and related drugs**

| Characteristic | AD cohort,  N=3,646 | non-AD cohort,  N=3,646 | P |
| --- | --- | --- | --- |
| Age at baseline, mean(95% CI) | 83.5 (83.3-83.7) | 83.5 (83.3-83.7) | matched |
| Age at first fracture during the follow-up | 85.6 (85.1-86.2) | 86.2 (85.5-86.8) | 0.22 |
| Age at first fracture before the follow-up | 78.3 (78.1-78.6) | 76.5 (76.2-76.8) | <0.001 |
| Sex |  |  |  |
| Men | 2958 (81.1) | 2958 (81.1) | matched |
| Women | 688 (18.9) | 688 (18.9) |  |
| Highest occupational social class before follow-up |  |  |  |
| Managerial/ professional | 620 (17.0) | 600 (16.5) | <0.001 |
| Office worker | 365 (10.0) | 346 (9.5) |  |
| Farming/forestry | 704 (19.3) | 790 (21.7) |  |
| Sales/industry/cleaning | 1479 (40.6) | 1330 (36.5) |  |
| Unknown | 459 (12.6) | 550 (15.1) |  |
| Did not respond | 19 (0.5) | 30 (0.8) |  |
| Hip replacement | 223 (6.2) | 143 (3.9) | <0.001 |
| Knee replacement | 10 (0.3) | 14 (0.4) | 0.41 |
| Cardiovascular disease | 1874 (51.4) | 1853 (50.8) | 0.62 |
| Stroke | 472 (12.9) | 455 (12.5) | 0.55 |
| Diabetes | 491 (13.5) | 414 (11.4) | 0.006 |
| Asthma/COPD | 353 (9.7) | 334 (9.2) | 0.44 |
| Cancer treatment | 54 (1.5) | 92 (2.5) | 0.001 |
| Any mental or behavioural disorder | 1302 (35.7) | 650 (17.8) | <0.001 |
| Any CVD medication | 3230 (88.6) | 3215 (88.2) | 0.58 |
| Drugs for obstructive airway diseases | 906 (24.8) | 890 (24.4) | 0.66 |
| Hormone therapy | 155 (4.3) | 141 (3.9) | 0.41 |
| Bisphosphonates | 1359 (37.3) | 1286 (35.3) | 0.08 |
| PPI | 1631 (44.7) | 1654 (45.4) | 0.59 |
| Anti-Parkinson drugs | 138 (3.8) | 168 (4.6) | 0.08 |
| Antiepileptics drugs | 463 (12.7) | 490 (13.4) | 0.35 |
| Antipsychotics | 852 (23.4) | 650 (17.8) | <0.001 |
| Antidepressants | 1554 (42.6) | 1237 (33.9) | <0.001 |
| BZDRs | 2005 (55.0) | 1968 (54.0) | 0.38 |
| Opioids | 2412 (66.2) | 2340 (64.2) | 0.08 |

**Table S2 Hip fracture risk factors in AD and non-AD cohorts (persons with previous fractures included, N=70,718 per cohort). Adjusted model is adjusted for occupational social class. COPD; chronic obstructive pulmonary disease; CVD cardiovascular disease; PPI proton pump inhibitors; BZDRs benzodiazepines and related drugs**

|  |  | AD cohort | | | Non-AD cohort | | |
| --- | --- | --- | --- | --- | --- | --- | --- |
| Risk factor | Category | IR/  100 PY | unadjusted HR  (95% CI) | adjusted HR  (95% CI) | IR/  100 PY | unadjusted HR (95% CI) | adjusted HR  (95% CI) |
| Age at baseline, increase per year | | | 1.07 (1.06-1.07) | 1.06 (1.06-1.07) | N.A | 1.11 (1.11-1.12) | 1.12 (1.11-1.12) |
| Hip fracture before follow-up | No | 2.1 | 1.00 (reference) | 1.00 (reference) | 0.90 | 1.00 (reference) | 1.00 (reference) |
|  | Yes | 4.1 | 1.88 (1.70-2.08) | 1.46 (1.32-1.61) | 2.71 | 3.02 (2.68-3.40) | 2.02 (1.79-2.28) |
| Sex | Men | 2.59 | 1.00 (reference) | 1.00 (reference) | 1.14 | 1.00 (reference) | 1.00 (reference) |
|  | Women | 1.63 | 0.63 (0.59-0.67) | 0.73 (0.68-0.78) | 0.66 | 0.58 (0.53-0.63) | 0.73 (0.66-0.80) |
| Highest occupational social class before follow-up | Managerial/ professional | 1.94 | 1.00 (reference) | 1.00 (reference) | 0.72 | 1.00 (reference) | N.A |
|  | Office worker | 2.67 | 1.37 (1.23-1.53) | 1.16 (1.04-1.29) | 1.07 | 1.49 (1.28-1.75) |  |
|  | Farming/forestry | 2.11 | 1.09 (1.00-1.19) | 0.92 (0.84-1.00) | 1.03 | 1.46 (1.28-1.66) |  |
|  | Sales/industry/cleaning | 2.21 | 1.14 (1.06-1.23) | 1.01 (0.94-1.09) | 1.02 | 1.43 (1.28-1.60) |  |
|  | Unknown | 3.30 | 1.70 (1.54-1.88) | 1.12 (1.01-1.25) | 1.61 | 2.29 (1.99-2.65) |  |
|  | Did not respond | 2.56 | 1.32 (0.97-1.79) | 1.13 (0.83-1.53) | 0.26 | 0.37 (0.25-0.54) |  |
| Hip replacement | No | 1.25 | 1.00 (reference) | 1.00 (reference) | 0.56 | 1.00 (reference) | 1.00 (reference) |
|  | Yes | 43.31 | 42.23 (39.86-44.74) | 39.10 (36.90-41.44) | 37.98 | 89.48 (82.41-97.17) | 68.29 (62.76-74.30) |
| Knee replacement | No | 2.22 | 1.00 (reference) | 1.00 (reference) | 0.95 | 1.00 (reference) | 1.00 (reference) |
|  | Yes | 60.56 | 27.81 (23.11-33.48) | 25.05 (20.80-30.17) | 39.56 | 45.24 (36.21-56.53) | 39.89 (31.89-49.89) |
| Cardiovascular disease | No | 2.26 | 1.00 (reference) | 1.00 (reference) | 0.90 | 1.00 (reference) | 1.00 (reference) |
|  | Yes | 2.28 | 1.01 (0.95-1.06) | 0.95 (0.90-1.00) | 1.06 | 1.18 (1.09-1.27) | 0.98 (0.91-1.06) |
| Stroke | No | 2.26 | 1.00 (reference) | 1.00 (reference) | 0.94 | 1.00 (reference) | 1.00 (reference) |
|  | Yes | 2.36 | 1.04 (0.95-1.15) | 1.04 (0.94-1.14) | 1.49 | 1.61 (1.43-1.81) | 1.38 (1.23-1.55) |
| Diabetes | No | 2.28 | 1.00 (reference) | 1.00 (reference) | 0.97 | 1.00 (reference) | 1.00 (reference) |
|  | Yes | 2.20 | 0.97 (0.89-1.05) | 1.03 (0.94-1.12) | 1.07 | 1.11 (0.99-1.25) | 1.14 (1.01-1.28) |
| Asthma/COPD | No | 2.26 | 1.00 (reference) | 1.00 (reference) | 0.98 | 1.00 (reference) | 1.00 (reference) |
|  | Yes | 2.33 | 1.03 (0.93-1.13) | 1.04 (0.95-1.15) | 1.01 | 1.03 (0.90-1.18) | 1.05 (0.92-1.20) |
| Cancer treatment | No | 2.27 | 1.00 (reference) | 1.00 (reference) | 0.97 | 1.00 (reference) | 1.00 (reference) |
|  | Yes | 2.18 | 0.96 (0.74-1.25) | 1.04 (0.80-1.35) | 1.51 | 1.54 (1.16-2.05) | 1.78 (1.34-2.36) |
| Any mental or behavioural disorder | No | 2.17 | 1.00 (reference) | 1.00 (reference) | 0.92 | 1.00 (reference) | 1.00 (reference) |
|  | Yes | 2.63 | 1.22 (1.14-1.29) | 1.16 (1.09-1.23) | 1.64 | 1.81 (1.62-2.01) | 1.67 (1.50-1.86) |
| Any CVD medication | No | 2.28 | 1.00 (reference) | 1.00 (reference) | 0.67 | 1.00 (reference) | 1.00 (reference) |
|  | Yes | 2.27 | 1.00 (0.92-1.07) | 0.85 (0.79-0.91) | 1.06 | 1.57 (1.41-1.75) | 1.05 (0.93-1.17) |
| Drugs for obstructive airway diseases | No | 2.29 | 1.00 (reference) | 1.00 (reference) | 0.97 | 1.00 (reference) | 1.00 (reference) |
|  | Yes | 2.21 | 0.96 (0.90-1.03) | 0.97 (0.91-1.04) | 1.00 | 1.02 (0.93-1.12) | 1.02 (0.93-1.12) |
| Hormone therapy | No | 231 | 1.00 (reference) | 1.00 (reference) | 1.01 | 1.00 (reference) | 1.00 (reference) |
|  | Yes | 1.74 | 0.75 (0.67-0.84) | 0.88 (0.78-0.99) | 0.55 | 0.54 (0.45-0.66) | 0.77 (0.63-0.95) |
| Bisphosphonates | No | 2.20 | 1.00 (reference) | 1.00 (reference) | 0.93 | 1.00 (reference) | 1.00 (reference) |
|  | Yes | 2.69 | 1.22 (1.13-1.31) | 0.98 (0.91-1.05) | 1.34 | 1.42 (1.28-1.58) | 1.04 (0.94-1.16) |
| PPI | No | 2.25 | 1.00 (reference) | 1.00 (reference) | 0.90 | 1.00 (reference) | 1.00 (reference) |
|  | Yes | 2.31 | 1.03 (0.97-1.09) | 0.98 (0.92-1.03) | 1.17 | 1.30 (1.20-1.40) | 1.16 (1.07-1.25) |
| Anti-Parkinson drugs | No | 2.25 | 1.00 (reference) | 1.00 (reference) | 0.96 | 1.00 (reference) | 1.00 (reference) |
|  | Yes | 2.98 | 1.33 (1.16-1.52) | 1.42 (1.24-1.62) | 1.82 | 1.88 (1.57-2.25) | 1.68 (1.40-2.01) |
| Antiepileptics drugs | No | 2.28 | 1.00 (reference) | 1.00 (reference) | 0.95 | 1.00 (reference) | 1.00 (reference) |
|  | Yes | 2.18 | 0.96 (0.87-1.05) | 0.99 (0.90-1.09) | 1.36 | 1.40 (1.24-1.59) | 1.30 (1.15-1.48) |
| Antipsychotics | No | 2.20 | 1.00 (reference) | 1.00 (reference) | 0.92 | 1.00 (reference) | 1.00 (reference) |
|  | Yes | 2.65 | 1.21 (1.13-1.29) | 1.13 (1.05-1.21) | 1.76 | 1.93 (1.73-2.16) | 1.54 (1.37-1.72) |
| Antidepressants | No | 2.22 | 1.00 (reference) | 1.00 (reference) | 0.89 | 1.00 (reference) | 1.00 (reference) |
|  | Yes | 2.37 | 1.07 (1.01-1.13) | 1.06 (1.00-1.12) | 1.36 | 1.53 (1.40-1.67) | 1.35 (1.24-1.47) |
| BZDRS | No | 2.09 | 1.00 (reference) | 1.00 (reference) | 0.80 | 1.00 (reference) | 1.00 (reference) |
|  | Yes | 2.50 | 1.19 (1.13-1.26) | 1.04 (0.99-1.10) | 1.29 | 1.61 (1.49-1.74) | 1.24 (1.14-1.34) |
| Opioids | No | 2.14 | 1.00 (reference) | 1.00 (reference) | 0.86 | 1.00 (reference) | 1.00 (reference) |
|  | Yes | 2.47 | 1.15 (1.09-1.21) | 1.02 (0.96-1.08) | 1.22 | 1.39 (1.28-1.50) | 1.11 (1.02-1.20) |

**Table S3 Predictors of mortality after hip fracture risk factors in AD and non-AD cohorts (persons with previous fractures included). Adjusted model is adjusted for occupational social class. COPD; chronic obstructive pulmonary disease; CVD cardiovascular disease; PPI proton pump inhibitors; BZDRs benzodiazepines and related drugs**

|  |  | AD cohort | | | Non-AD cohort | | |
| --- | --- | --- | --- | --- | --- | --- | --- |
| Risk factor | Category | mortality /100 PY | unadjusted HR  (95% CI) | adjusted HR  (95% CI) | mortality  /100 PY | unadjusted HR (95% CI) | adjusted HR  (95% CI) |
| Age at baseline, increase per year | | | 1.07 (1.06-1.07) | 1.06 (1.06-1.07) | N.A | 1.11 (1.11-1.12) | 1.12 (1.11-1.12) |
| Hip fracture before follow-up | No | 29.2 | 1.00 (reference) | 1.00 (reference) | 22.9 | 1.00 (reference) | 1.00 (reference) |
|  | Yes | 27.9 | 0.96 (0.83-1.10) | 0.92 (0.80-1.06) | 18.8 | 0.83 (0.68-1.00) | 0.87 (0.71-1.05) |
| Sex | Men | 24.9 | 1.00 (reference) | 1.00 (reference) | 19.0 | 1.00 (reference) | 1.00 (reference) |
|  | Women | 47.2 | 1.89 (1.74-2.05) | 2.09 (1.91-2.28) | 36.8 | 1.96 (1.71-2.23) | 2.33 (2.03-2.67) |
| Highest occupational social class before follow-up | Managerial/ professional | 29.4 | 1.00 (reference) | 1.00 (reference) | 22.2 | 1.00 (reference) | N.A |
|  | Office worker | 25.8 | 0.88 (0.75-1.03) | 1.04 (0.89-1.22) | 21.9 | 0.97 (0.75-1.27) |  |
|  | Farming/forestry | 30.6 | 1.04 (0.91-1.19) | 1.02 (0.90-1.17) | 21.4 | 0.96 (0.78-1.18) |  |
|  | Sales/industry/cleaning | 29.7 | 1.01 (0.90-1.13) | 1.07 (0.96-1.20) | 23.3 | 1.04 (0.86-1.25) |  |
|  | Unknown | 28.2 | 0.96 (0.83-1.11) | 1.04 (0.90-1.20) | 23.8 | 1.08 (0.86-1.35) |  |
|  | Did not respond | 23.6 | 0.80 (0.51-1.24) | 0.97 (0.62-1.50) | 23.3 | 1.03 (0.53-2.02) |  |
| Hip replacement | No | 29.36 | 1.00 (reference) | 1.00 (reference) | 22.74 | 1.00 (reference) | 1.00 (reference) |
|  | Yes | 24.56 | 0.84 (0.68-1.03) | 0.80 (0.65-0.98) | 20.98 | 0.93 (0.69-1.24) | 1.19 (0.88-1.59) |
| Knee replacement | No | 29.51 | 1.00 (reference) | 1.00 (reference) | 22.81 | 1.00 (reference) | 1.00 (reference) |
|  | Yes | 19.62 | 0.66 (0.52-0.86) | 0.73 (0.57-0.94) | 17.53 | 0.77 (0.51-1.15) | 0.91 (0.60-1.36) |
| Cardiovascular disease | No | 25.9 | 1.00 (reference) | 1.00 (reference) | 19.7 | 1.00 (reference) | 1.00 (reference) |
|  | Yes | 32.5 | 1.25 (1.16-1.35) | 1.23 (1.14-1.33) | 25.1 | 1.27 (1.12-1.44) | 1.29 (1.14-1.47) |
| Stroke | No | 28.6 | 1.00 (reference) | 1.00 (reference) | 21.6 | 1.00 (reference) | 1.00 (reference) |
|  | Yes | 36.0 | 1.26 (1.10-1.44) | 1.20 (1.04-1.37) | 37.3 | 1.72 (1.41-2.08) | 1.67 (1.37-2.02) |
| Diabetes | No | 28.5 | 1.00 (reference) | 1.00 (reference) | 22.6 | 1.00 (reference) | 1.00 (reference) |
|  | Yes | 33.0 | 1.18 (1.05-1.32) | 1.25 (1.11-1.40) | 23.2 | 1.03 (0.86-1.23) | 1.14 (0.95-1.36) |
| Asthma/COPD | No | 28.8 | 1.00 (reference) | 1.00 (reference) | 22.7 | 1.00 (reference) | 1.00 (reference) |
|  | Yes | 33.5 | 1.17 (1.02-1.33) | 1.19 (1.04-1.35) | 22.4 | 0.97 (0.79-1.20) | 1.08 (0.88-1.34) |
| Cancer treatment | No | 29.2 | 1.00 (reference) | 1.00 (reference) | 23.3 | 1.00 (reference) | 1.00 (reference) |
|  | Yes | 22.4 | 0.77 (0.51-1.16) | 0.84 (0.56-1.26) | 26.4 | 0.95 (0.61-1.49) | 1.45 (0.93-2.27) |
| Any mental or behavioural disorder | No | 28.2 | 1.00 (reference) | 1.00 (reference) | 22.1 | 1.00 (reference) | 1.00 (reference) |
|  | Yes | 31.4 | 1.11 (1.03-1.21) | 1.18 (1.08-1.28) | 27.6 | 1.26 (1.04-1.53) | 1.33 (1.09-1.62) |
| Any CVD medication | No | 23.6 | 1.00 (reference) | 1.00 (reference) | 17.6 | 1.00 (reference) | 1.00 (reference) |
|  | Yes | 30.0 | 1.27 (1.12-1.44) | 1.29 (1.14-1.46) | 23.2 | 1.31 (1.04-1.65) | 1.34 (1.06-1.68) |
| Drugs for obstructive airway diseases | No | 28.2 | 1.00 (reference) | 1.00 (reference) | 22.4 | 1.00 (reference) | 1.00 (reference) |
|  | Yes | 35.0 | 1.24 (1.12-1.37) | 1.18 (1.06-1.31) | 23.8 | 1.05 (0.90-1.23) | 1.13 (0.96-1.32) |
| Hormone therapy | No | 29.7 | 1.00 (reference) | 1.00 (reference) | 23.7 | 1.00 (reference) | 1.00 (reference) |
|  | Yes | 18.8 | 0.63 (0.50-0.80) | 0.83 (0.66-1.04) | 11.7 | 0.46 (0.27-0.76) | 0.69 (0.41-1.16) |
| Bisphosphonates | No | 29.5 | 1.00 (reference) | 1.00 (reference) | 23.2 | 1.00 (reference) | 1.00 (reference) |
|  | Yes | 27.4 | 0.93 (0.83-1.03) | 1.04 (0.94-1.16) | 20.1 | 0.86 (0.73-1.02) | 1.04 (0.87-1.24) |
| PPI | No | 20.5 | 1.00 (reference) | 1.00 (reference) | 21.0 | 1.00 (reference) | 1.00 (reference) |
|  | Yes | 25.6 | 1.23 (1.14-1.33) | 1.24 (1.15-1.35) | 28.8 | 1.24 (1.09-1.40) | 1.29 (1.14-1.46) |
| Anti-Parkinson drugs | No | 29.16 | 1.00 (reference) | 1.00 (reference) | 22.55 | 1.00 (reference) | 1.00 (reference) |
|  | Yes | 29.22 | 1.00 (0.85-1.18) | 1.02 (0.87-1.21) | 24.33 | 1.09 (0.84-1.40) | 1.12 (0.87-1.45) |
| Antiepileptics drugs | No | 28.9 | 1.00 (reference) | 1.00 (reference) | 22.9 | 1.00 (reference) | 1.00 (reference) |
|  | Yes | 31.4 | 1.09 (0.96-1.23) | 1.11 (0.98-1.26) | 20.8 | 0.91 (0.74-1.10) | 1.00 (0.82-1.22) |
| Antipsychotics | No | 28.1 | 1.00 (reference) | 1.00 (reference) | 21.5 | 1.00 (reference) | 1.00 (reference) |
|  | Yes | 31.2 | 1.11 (1.03-1.20) | 1.17 (1.08-1.26) | 31.0 | 1.46 (1.24-1.72) | 1.48 (1.25-1.74) |
| Antidepressants | No | 30.6 | 1.00 (reference) | 1.00 (reference) | 21.6 | 1.00 (reference) | 1.00 (reference) |
|  | Yes | 27.4 | 0.90 (0.83-0.97) | 0.98 (0.90-1.06) | 25.7 | 1.19 (1.04-1.36) | 1.27 (1.11-1.46) |
| BZDRS | No | 28.2 | 1.00 (reference) | 1.00 (reference) | 20.5 | 1.00 (reference) | 1.00 (reference) |
|  | Yes | 30.1 | 1.07 (0.99-1.16) | 1.07 (0.99-1.15) | 25.1 | 1.23 (1.09-1.39) | 1.20 (1.06-1.36) |
| Opioids | No | 25.5 | 1.00 (reference) | 1.00 (reference) | 18.3 | 1.00 (reference) | 1.00 (reference) |
|  | Yes | 32.5 | 1.28 (1.18-1.39) | 1.27 (1.17-1.38) | 27.2 | 1.46 (1.29-1.66) | 1.52 (1.34-1.73) |
